# Integrating Neutrophil‐To‐Albumin Ratio and Triglycerides: A Novel Indicator for Predicting Spontaneous Hemorrhagic Transformation in Acute Ischemic Stroke Patients

**DOI:** 10.1111/cns.70133

**Published:** 2024-12-17

**Authors:** Jiajia Bao, Mengmeng Ma, Kongyuan Wu, Jian Wang, Muke Zhou, Jian Guo, Ning Chen, Jinghuan Fang, Li He

**Affiliations:** ^1^ The Neurology Department of West China Hospital Sichuan University Chengdu China

**Keywords:** AIS, NAR, NATG, spontaneous hemorrhagic transformation (sHT), triglycerides (TGs)

## Abstract

**Background:**

Hemorrhagic transformation (HT) is a tragic complication of acute ischemic stroke (AIS), with spontaneous HT (sHT) occurring even without reperfusion therapies. Despite evidence suggesting that several inflammation biomarkers are closely related to HT, its utility in sHT risk stratification remains unclear. This study aimed to identify and integrate effective inflammatory biomarkers associated with sHT and to develop a novel nomogram model for the early detection of sHT.

**Methods:**

We conducted a retrospective observational cohort study of AIS patients receiving conventional medical treatment solely from March 2022 to March 2023, using a prospectively maintained database. All patients underwent CT follow‐up within 7 days after admission, with sHT occurrence within this period as the outcome. Data on demographics, clinical information, laboratory results, and imaging were collected. The cohort was divided into training and validation sets (7:3). Least absolute shrinkage and selection operator (LASSO) regression selected inflammatory biomarkers for a novel index. Univariable and multivariable logistic regressions were conducted to identify independent sHT risk factors. Receiver operating characteristic (ROC) analysis determined optimal cut‐off values for continuous factors. A nomogram was developed and validated internally and externally. Predictive accuracy was assessed using the area under the ROC curve (AUC) and calibration plots. Decision curve analysis (DCA) evaluated clinical usefulness.

**Results:**

Of 803 AIS patients, 325 were included in the final analysis. sHT was found in 9.5% (31 patients). Training (*n* = 228) and validation (*n* = 97) cohorts showed no significant demographic or clinical differences. LASSO regression integrated neutrophil‐to‐albumin ratio (NAR) and triglycerides (TGs) into a novel index—NATG. Independent sHT risk factors included baseline National Institute of Health Stroke Scale (NIHSS) (OR = 1.09, 95% CI (1.02, 1.16), *p* = 0.0095), NATG (OR = 1534.87, 95% CI (5.02, 469638.44), *p* = 0.0120), D‐dimer (DD) (OR = 1.12, 95% CI (1.01, 1.25), *p* = 0.0249), and total cholesterol (TC) (OR = 1.01, 95% CI (1.00, 1.01), *p* = 0.0280), with their respective optimal cut‐off values being 13, 0.059, 0.86, and 3.6. These factors were used to develop the nomogram in the training cohort, which achieved an AUC of 0.804 (95% CI, 0.643–0.918) in the training cohort and 0.713 (95% CI, 0.499–0.868) in the validation cohort, demonstrating consistent calibration. DCA confirmed the nomogram's clinical applicability in both cohorts.

**Conclusions:**

A novel indicator combining NAR and TG is positively associated with sHT in AIS patients. The constructed nomogram, integrating this novel indicator with other risk factors, provides a valuable tool for identifying sHT risk, aiding in clinical decision‐making.

## Graphical abstract

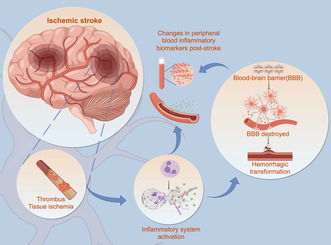

## 1 Introduction

Hemorrhagic transformation (HT) is a natural and spontaneous tragic complication of acute ischemic stroke (AIS), which is related to increased mortality and a worse functional outcome. HT occurs in 3%–40% of AIS patients [1, 2], exacerbated by reperfusion with alteplase (recombinant tissue plasminogen activator) or endovascular therapy (EVT). However, most AIS patients did not receive thrombolysis and thrombectomy in the real world, and the overall frequency of HT in those AIS patients was 0.4%–10.3% [3], which has been regarded as spontaneous HT (sHT) [4, 5].

Identifying early predictive risk factors before HT occurs has an effect on improving functional outcomes of AIS patients [6]. Accordingly, it is important to evaluate the prognostic risk factors for HT [6]. National Institute of Health Stroke Scale (NIHSS) on admission, medicine treatment (anticoagulant or antithrombotic treatment, and statin use), and collateral condition were associated with postthrombectomy and thrombolysis HT as previously reported [7, 8, 9, 10]. Moreover, accumulating evidence suggested that inflammation plays an important role in the process of HT after AIS [5]. Several combined inflammatory biomarkers, such as NLR, SII, and LMR, have been reported as potential risk factors for postthrombolysis and postthrombectomy HT [1, 11, 12, 13]. However, whether these inflammatory biomarkers could enable accurately stratify the risk of sHT remains unknown [14].

In this present study, we aimed to identify effective prognostic inflammatory biomarkers associated with the occurrence of sHT and develop a new nomogram model based on inflammatory biomarkers, collateral status, and other clinical risk factors. Our research could aid in the early detection of sHT and guide its clinical management strategies.

## 2 Methods

### 2.1 Ethics Statement

The study was conducted in compliance with the transparent reporting of a multivariable prediction model for individual prognosis or diagnosis (TRIPOD) statement [15]. The Ethical Review Committee of the West China Hospital of Sichuan University approved this research (No. 2020(069)). As this is a retrospective study that collects deidentified data, the requirement of obtaining informed consent was waived.

### 2.2 Study Design and Population

We performed a retrospective observational cohort of patients with AIS from March 2022 to March 2023 from a consecutively collected and prospectively maintained database in the Department of Neurology of our hospital. The diagnosis of AIS was based on the World Health Organization stroke diagnostic criteria and confirmed with evidence of neuroimaging, including magnetic resonance imaging (MRI) or computed tomography (CT). Patients were eligible for the study if they met the following criteria: (1) aged over 18 years old and admitted to the hospital within 7 days after onset of symptoms [16, 17, 18], (2) patients met the AIS diagnostic criteria based on WHO, and confirmed with the evidence of neuroimaging; (3) received conventional medication treatment alone without reperfusion therapies such as intravenous thrombolysis or endovascular thrombectomy; and (4) underwent baseline laboratory investigation within 24 h after admission. Exclusion criteria were as follows: (1) pre‐AIS modified Rankin scale score (mRS) was over 2; (2) missing baseline or follow‐up brain imaging; (3) infection within 2 weeks prior to AIS onset; (4) patients with cerebral hemorrhage, brain tumors, other severe space‐occupying effect diseases, cerebral aneurysm, and cerebrovascular malformation; (5) patients with hematological disorders, and severe hepatic or renal dysfunction, and (6) patients with unavailable or incomplete clinical and imaging information.

**FIGURE 1:**
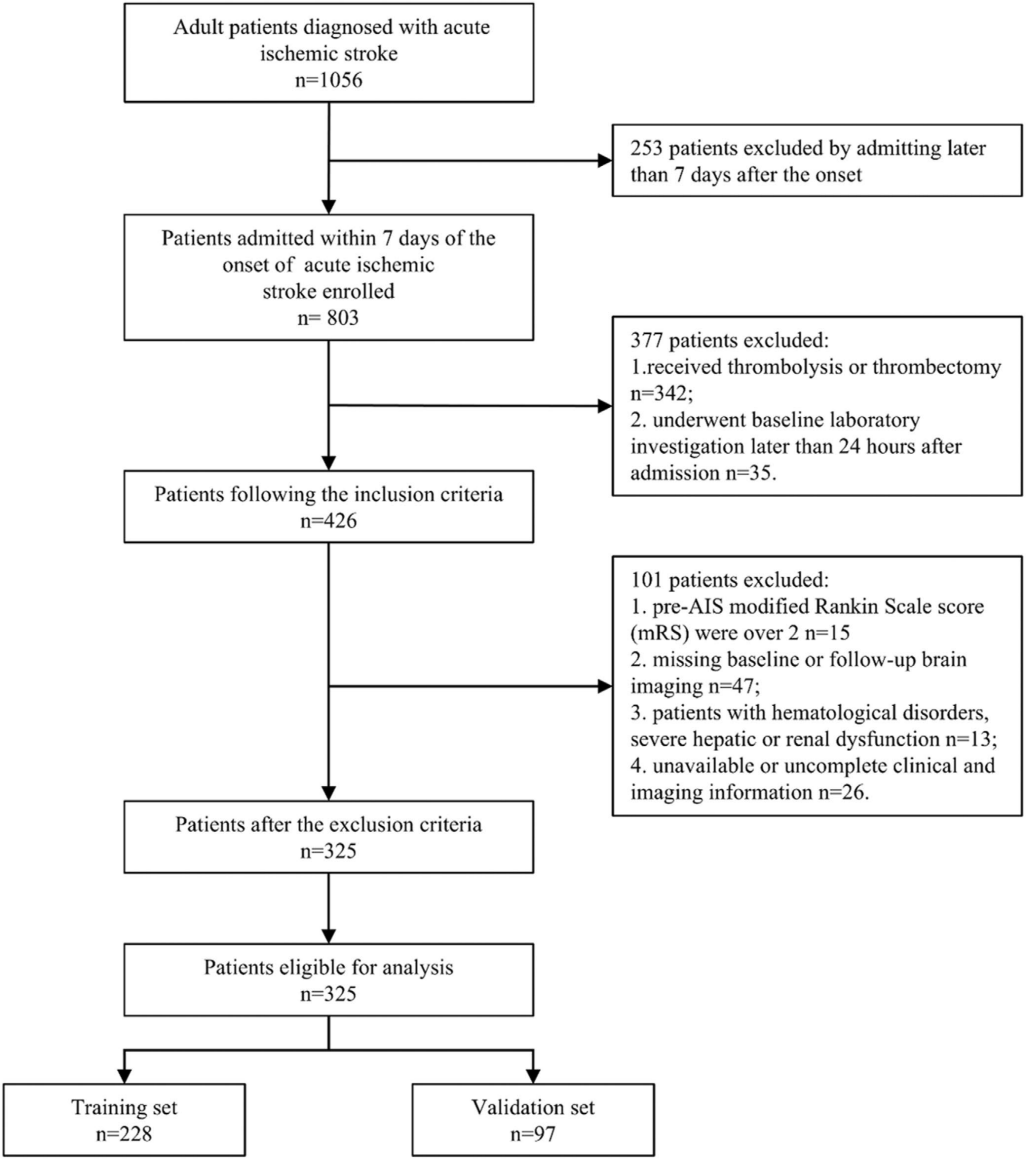
Flow diagram of patients in this study. AIS = acute ischemic stroke.

Finally, a total of 325 patients were met the criteria and included in final analysis. The flowchart of study population selection and screening is shown in Figure 1. All patients underwent CT follow‐up within 7 days after admission and immediately in case of a worsening condition.

### 2.3 Clinical Data and Outcome Assessment

We collected the demographic characteristics, vascular risk factors, medical history, laboratory examination data, imaging information, and therapy treatment (including prior and post‐AIS onset) at admission and during hospitalization.

BMI was calculated by weight (kilograms) divided by the square of the height (meters). The definitions of hypertension, diabetes mellitus, atrial fibrillation, and coronary heart disease are in accordance with WHO criteria. The collateral status was evaluated through the regional leptomeningeal collateral (rLMC) scores in accordance with previous study [19]. The severity of stroke was assessed by the NIHSS at admission and the stroke subtypes were classified according to the Trial of Org 10172 in Acute Stroke Treatment (TOAST) classification, as described previously [20].

All blood venous samples, including complete blood count (CBC), biochemistry panel, coagulation, and thyroid functions were collected and tested within 24 h of admission in accordance with the standard institutional guidelines [11, 12, 13]. The inflammatory parameters derived by CBC were also calculated. The neutrophil‐to‐lymphocyte ratio (NLR), systemic immune‐inflammation index (SII), platelet–lymphocyte ratio (PLR), lymphocyte‐to‐monocyte ratio (LMR), neutrophil‐to‐albumin ratio (NAR), prognostic nutritional index (PNI), derived neutrophil‐to‐lymphocyte ratio (dNLR), albumin–bilirubin (ALBI), platelet–albumin–bilirubin (PALBI), and low‐density lipoprotein cholesterol‐to‐lymphocyte ratio were defined as previous studies described [18, 21, 22].

The primary outcome was defined as the occurrence of sHT within 7 days after admission based on the initial and follow‐up brain imaging [23]. HT was defined as any type of intracranial hemorrhage (ICH) observed on follow‐up CT, which was not observed on initial CT imaging according to the European Cooperative Acute Stroke Study (ECASS) II criteria [24]. All enrolled patients underwent at least one follow‐up neuroimaging (CT and/or MRI) within 7 days after admission and immediately in case of a worsening condition. Two neurologists who were blinded to the patients' information independently reviewed and evaluated the neuroimages. If there were any controversial diagnoses, the images would be reviewed by a third neurologist. In line with previous studies, the HT observed in patients who received conventional medical therapy alone was regarded as sHT in this study [4, 25]. Due to the limitation in sample size, the subtypes of HT were not categorized.

### 2.4 Development of the Nomogram

#### 2.4.1 Variables Selection and Nomogram Model Construction

We randomly divided the whole cohort into the nomogram development set and validation set at a 7:3 ratio. The training cohort was used to build the nomogram. Considering the possibility of multicollinearity among inflammatory biomarkers, we initially conducted the Least Absolute Shrinkage and Selection Operator (LASSO) analysis with 10‐fold cross‐validation to exclude the relatively unimportant variables and select important variables by incorporating an L1 regularization penalty, thereby simplifying the model structure and preventing overfitting. Inflammatory biomarkers with nonzero coefficients in the LASSO regression were combined to develop a novel index [22]. Sensitivity analysis involving stratified analysis and interaction test was also conducted to evaluate whether the inflammatory biomarkers selected by LASSO exhibit potential interactive or synergistic effects. Subsequently, univariable and multivariable logistic regression were used to identify independent risk factors for sHT. For the multivariable analysis, variables that showed a *p* < 0.05 in the univariate analysis and were clinically relevant were included.

The optimal cut‐off value of these identified continuous factors for predicting HT of AIS patients with conventional medical treatment alone was determined through the receiver operating characteristic (ROC) curves, which were then classified as categorical variables according to the Youden index, respectively.

The nomogram of the prediction model based on the risk factors selected through multivariable logistic regression was established using stepwise logistic regression analyses. The *F* probability thresholds were set at 0.05 for entry and 0.10 for removal. The effect estimates of these selected factors were presented as the odds ratios (OR) with 95% confidence interval (CI) and *p* values.

#### 2.4.2 Validation and Evaluation of the Nomogram

The evaluation of the effect of the nomogram by internal validation in the training cohort bootstrapping validation (500 bootstrap resamples) and external validation in the validation cohort. Then, we conducted the area under the ROC curve (AUC) to evaluate the predictive accuracy of the nomogram. The calibration curves were plotted to assess the calibration of the nomogram in both the training and the validation cohort by comparing the predictive sHT occurrence with actual observed risk. For the clinical usefulness of our nomogram, we further assessed the decision curve analysis (DCA) to quantify the net benefit across a range of threshold probabilities.

### 2.5 Statistical Analyses

All patients were randomly assigned into two cohorts in a ratio of 7:3: the training cohort and the validation cohort. Descriptive statistics and univariate comparisons were conducted in both cohorts. Continuous data were expressed as mean ± standard deviation (SD) or median (interquartile range [IQR]) by using Student's *t*‐test or Mann–Whitney *U*‐test. Categorical variables were shown as absolute values (percentages) through Chi‐square test or Fisher's exact test. The LASSO regression was used to select important inflammatory biomarkers. And the univariable and multivariable logistic regression were applied to determine the independent risk factors of sHT occurrence in AIS patients receiving conventional medical treatment. All statistical analyses were two tailed, and *p* < 0.05 was considered statistically significant. SPSS 25.0 (IBM, Armonk, NY, United States) and R software (version 4.0.2; R Foundation for Statistical Computing, Vienna, Austria) were used for all statistical analyses.

## 3 Results

### 3.1 Baseline Characteristics of Participants

From 803 patients with AIS, we identified 325 patients with AIS receiving conventional medical treatment solely who met our study criteria and enrolled our final analysis (Figure 1). A total of 9.5% (31 patients) sHT were found among total cohort. Of these patients, the average age was 69.1 ± 14.2 years, and 50.85% (300 patients) were male. All patients were randomly assigned to the training cohort (7/10 of the sample, *n* = 228) and the validation cohort (3/10 of the sample, *n* = 97). There were 22 (9.6%) patients and 9 (9.3%) patients occurred sHT in the training cohort and validation cohort, respectively. The demographic and clinical characteristics of the total patients and the comparison of the training and validating cohorts are presented in Table 1. No significant differences in demographics and clinical characteristics were found between both cohorts.

**TABLE 1:** Demographic and clinical characteristics of patients in the training and validation cohorts (*n* = 325).

	All patients (*n* = 325)	Training cohort (*n* = 228)	Validation cohort (*n* = 97)	*p*
Age, mean ± SD, year	69.1 ± 14.2	69.9 ± 13.1	67.2 ± 16.3	0.124
Gender, *n* (%)				0.126
Female	145 (44.6%)	108 (47.4%)	37 (38.1%)	
Male	300 (50.85%)	120 (52.6%)	60 (61.9%)	
Blood pressure, mean ± SD, mmHg				
SBP	149.4 ± 25.2	148.3 ± 25.0	151.8 ± 25.7	0.258
DBP	85.1 ± 15.0	83.8 ± 15.1	88.2 ± 14.5	0.015*
BMI, mean ± SD, kg/m^2^	23.7 ± 3.2	23.9 ± 3.2	23.5 ± 3.1	0.387
Preexisting conditions, *n* (%)				
Hypertension	187 (57.5%)	136 (59.6%)	51 (52.6%)	0.238
Diabetes mellitus	54 (16.6%)	34 (14.9%)	20 (20.6%)	0.206
Atrial fibrillation	82 (25.2%)	59 (25.9%)	23 (23.7%)	0.681
Coronary heart disease	42 (12.9%)	30 (13.2%)	12 (12.4%)	0.847
Current smoking	119 (36.6%)	83 (36.4%)	36 (37.1%)	0.903
Medication history, *n* (%)				
Antiplatelet, *n* (%)	39 (12.0%)	26 (11.4%)	13 (13.4%)	0.612
Anticoagulant, *n* (%)	39 (12.0%)	29 (12.7%)	10 (10.3%)	0.541
Statins, *n* (%)	33 (10.2%)	25 (11.0%)	8 (8.2%)	0.458
Clinical variables				
Baseline NIHSS, median (IQR)	316.5 ± 400.3	10.1 ± 7.7	10.0 ± 7.8	0.889
TOAST				0.140
LAO, *n* (%)	112 (34.5%)	84 (36.8%)	28 (28.9%)	
CE, *n* (%)	135 (41.5%)	96 (42.1%)	39 (40.2%)	
SAO, *n* (%)	20 (6.2%)	12 (5.3%)	8 (8.2%)	
OE, *n* (%)	10 (3.1%)	4 (1.8%)	6 (6.2%)	
UE, *n* (%)	48 (14.8%)	32 (14.0%)	16 (16.5%)	
Onset to admission time, median (IQR), min	10.1 ± 7.7	305.7 ± 277.7	341.6 ± 597.2	0.461
rLMC, median (IQR)	13.9 ± 4.8	13.9 ± 4.7	13.8 ± 5.1	0.835
Laboratory parameters, mean ± SD				
NLR	6.2 ± 5.7	6.2 ± 5.8	6.3 ± 5.5	0.984
SII	1037.7 ± 1277.0	1066.7 ± 1424.9	970.2 ± 839.9	0.541
PLR	150.6 ± 99.9	155.1 ± 103.2	140.0 ± 91.3	0.222
LMR	5.2 ± 20.4	5.7 ± 24.3	4.2 ± 4.1	0.563
NAR	0.2 ± 0.1	0.2 ± 0.2	0.2 ± 0.1	0.679
PNI	42.1 ± 5.0	41.9 ± 5.5	42.6 ± 3.8	0.258
DNLR	−1.1 ± 11.0	−0.6 ± 11.9	−2.5 ± 8.2	0.143
ALBI	−2.9 ± 0.4	−2.8 ± 0.5	−2.9 ± 0.3	0.32
PALBI	−1.2 ± 0.3	−1.2 ± 0.3	−1.2 ± 0.2	0.774
LDL‐C/lymphocyte	2.3 ± 1.4	2.3 ± 1.4	2.2 ± 1.6	0.551
Platelet, ×109/L	167.9 ± 65.4	170.9 ± 67.9	160.7 ± 59.0	0.196
Hb, g/L	133.4 ± 20.0	132.7 ± 19.7	135.1 ± 20.8	0.323
INR	1.3 ± 4.7	1.0 ± 0.2	1.9 ± 8.5	0.123
DD, mg/L	2.1 ± 4.1	2.0 ± 4.3	2.1 ± 3.9	0.853
TC, mmol/L	7.3 ± 37.8	8.7 ± 45.3	4.2 ± 1.1	0.338
TG, mmol/L	2.1 ± 9.9	2.3 ± 11.9	1.7 ± 1.2	0.625
LDL, mmol/L	2.5 ± 1.0	2.5 ± 1.0	2.5 ± 0.9	0.697
HDL, mmol/L	1.6 ± 4.4	1.4 ± 0.5	2.1 ± 8.0	0.168
Serum glucose, mmol/L	10.6 ± 47.1	11.7 ± 56.3	8.0 ± 2.8	0.525
Albumin, g/L	41.9 ± 5.1	41.7 ± 5.4	42.4 ± 4.1	0.290
Uric acid, μmol/L	363.5 ± 118.2	364.2 ± 122.2	361.9 ± 109.2	0.876
Creatinine, μmol/L	78.4 ± 30.8	78.1 ± 32.2	79.2 ± 27.4	0.784
Treatment after admission, *n* (%)				
Antiplatelet	274 (84.3%)	190 (83.3%)	84 (86.6%)	0.459
Anticoagulant	55 (16.9%)	38 (16.7%)	17 (17.5%)	0.85
Statins	289 (88.9%)	203 (89.0%)	86 (88.7%)	0.921
Antihypertensives	134 (41.2%)	93 (40.8%)	41 (42.3%)	0.804
HT, *n* (%)	31 (9.5%)	22 (9.6%)	9 (9.3%)	0.917

Abbreviations: ALBI = albumin–bilirubin; BMI=body mass index; CE = cardioembolic; DBP=diastolic blood pressure; DD=D‐Dimer; dNLR = derived neutrophil‐to‐lymphocyte ratio; HDL = high‐density lipoprotein; HT = hemorrhagic transformation; INR = international normalized ratio; IQR = interquartile range; LAO = large vessels atherosclerosis; LDL = low‐density lipoprotein; LMR = lymphocyte‐to‐monocyte ratio; NAR = neutrophil‐to‐albumin ratio; NATG = NAR–triglyceride ratio; NIHSS = National Institute of Health Stroke Scale; NLR = neutrophil‐to‐lymphocyte ratio; OE = other etiology; PALBI = platelet–albumin–bilirubin; PLR = platelet–lymphocyte ratio; PNI = prognostic nutritional index; rLMC = regional leptomeningeal score; SAO=small‐artery occlusion; SBP=systolic blood pressure; SD=Standard deviation; SII = systemic immune‐inflammation index; TC = total cholesterol; TG = triglyceride; TOAST = Trial of ORG 10172 in Acute Stroke Treatment; UE = undetermined etiology.

* *p* value less than 0.05.

### 3.2 LASSO Regression for Variables Selection

**FIGURE 2:**
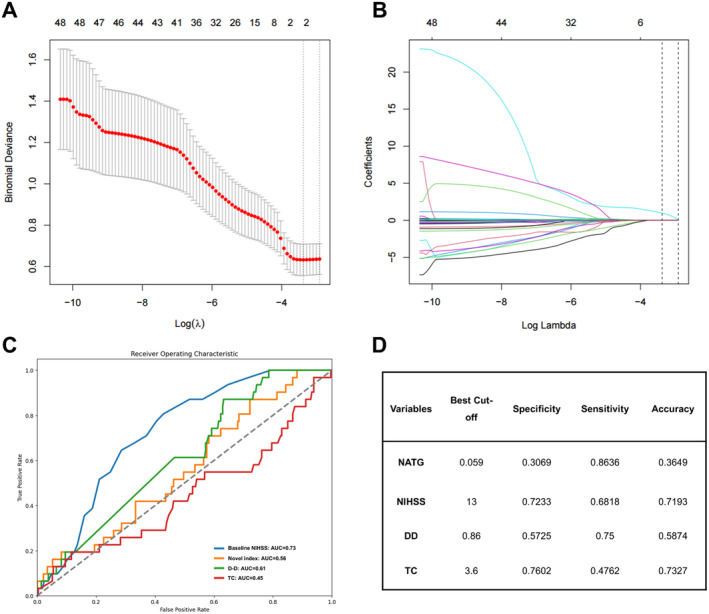
LASSO regression was used to select potential factors and develop a novel laboratory index, while ROC curves were used to evaluate the predictive value and determine the optimal cut‐off value of independent risk factors. (A) Partial likelihood deviance curve (binomial deviance). (B) Coefficient profile plot. (C) ROC curves. (D) Optimal cut‐off value. AUC = area under the ROC curve; DD=D‐dimer; LASSO = Least absolute shrinkage and selection operator; NATG = NAR–triglyceride ratio; NIHSS=National Institute of Health Stroke Scale; ROC = receiver operating characteristic; TC = total cholesterol.

We performed the LASSO regression to select important variables from 25 inflammatory biomarkers. Figure 2A shows the curve for partial likelihood deviance (binomial deviance), while Figure 2B depicts the coefficient profile plot. The optimal lambda (*λ*) value, determined at 1 standard error (1 se), was 0.0541 [log (lambda.1 se) = −2.9163], leading to the selection of NAR and triglyceride (TG) as significant factors. These were then incorporated into a novel index, defined as NATG, using the formula: NATG = 0.56 * NAR + 0.0025 * TG. Moreover, the sensitivity analysis indicated that NAR and TG had no interactive effect (Table S1).

### 3.3 Independent Predictors in the Training Cohort

To identify the independent factors related to sHT, univariable and multivariable logistic regression analyses were conducted in the training cohort, as shown in Table 2. In the univariate logistic regression analysis, gender, prior‐stroke anticoagulant use, baseline NIHSS, NATG, and statin use at admission were significantly associated with sHT in training cohort. After adjusting for potential confounders (Model 1: adjusted for age and gender; and Model 2: adjusted for age, gender, baseline NIHSS, TOAST, anticoagulant history, statins after admission, and antihypertension after admission), baseline NIHSS (OR = 1.09, 95% CI (1.02, 1.16), *p* = 0.0095), NATG (OR = 1534.87, 95% CI (5.02, 469638.44), *p* = 0.0120), D‐dimer (DD) (OR = 1.12, 95% CI (1.01, 1.25), *p* = 0.0249), total cholesterol (TC) (OR = 1.01, 95% CI (1.00, 1.01), *p* = 0.0280) were independent risk factors for the sHT (Table 2).

**TABLE 2:** Univariate and multivariate logistic regression analysis of prognostic factors for spontaneous hemorrhage transformation in AIS patients in training set.

Characteristics	Univariate regression	Multivariate regression	
	OR (95% CI), *p*	Model 1^a^	Model 2^b^
		OR (95% CI), *p*	OR (95% CI), *p*
Age, year	1.01 (0.98, 1.05) 0.5673	1.00 (0.97, 1.04) 0.8900	0.99 (0.95, 1.03) 0.6715
Gender, male	0.38 (0.15, 0.98) 0.0456	0.39 (0.15, 1.01) 0.0537	0.50 (0.16, 1.55) 0.2312
SBP, mmHg	1.00 (0.98, 1.02) 0.8013	1.00 (0.98, 1.02) 0.7033	1.00 (0.98, 1.02) 0.8477
DBP, mmHg	1.01 (0.98, 1.04) 0.6111	1.01 (0.98, 1.04) 0.5441	1.02 (0.99, 1.05) 0.3266
BMI, kg/m^2^	1.04 (0.85, 1.28) 0.7008	1.04 (0.85, 1.27) 0.7237	0.93 (0.71, 1.20) 0.5688
Preexisting conditions			
Hypertension	0.79 (0.33, 1.92) 0.6083	0.67 (0.26, 1.73) 0.4045	0.43 (0.14, 1.37) 0.1540
Diabetes mellitus	1.80 (0.61, 5.24) 0.2846	1.65 (0.56, 4.92) 0.3649	1.88 (0.56, 6.38) 0.3091
Atrial fibrillation	1.74 (0.69, 4.38) 0.2419	1.36 (0.51, 3.65) 0.5358	0.55 (0.15, 2.04) 0.3681
Coronary heart disease	1.54 (0.48, 4.90) 0.4662	1.27 (0.38, 4.20) 0.6977	0.77 (0.21, 2.88) 0.7018
Current smoking	0.48 (0.17, 1.36) 0.1682	0.84 (0.23, 3.07) 0.7901	1.21 (0.28, 5.16) 0.7987
Medication history	0.87 (0.46, 1.64) 0.6718	0.58 (0.26, 1.27) 0.1714	0.66 (0.26, 1.63) 0.3634
Antiplatelet	1.26 (0.34, 4.58) 0.7294	1.14 (0.31, 4.20) 0.8487	0.88 (0.21, 3.65) 0.8592
Anticoagulant	2.98 (1.06, 8.39) 0.0382*	2.65 (0.93, 7.57) 0.0691	1.80 (0.53, 6.09) 0.3465
Statins	1.96 (0.61, 6.33) 0.2619	2.27 (0.68, 7.59) 0.1821	2.18 (0.57, 8.29) 0.2543
Clinical variables			
Baseline NIHSS	1.09 (1.04, 1.15) 0.0013*	1.09 (1.03, 1.15) 0.0032*	1.09 (1.02, 1.16) 0.0095*
TOAST			
LAO, *n* (%)	—	—	—
CE, *n* (%)	3.13 (0.98, 10.01) 0.0541	2.36 (0.70, 7.94) 0.1649	1.08 (0.28, 4.23) 0.9116
SAO, *n* (%)	0.00 (0.00, Inf) 0.9898	0.00 (0.00, Inf) 0.9898	0.00 (0.00, Inf) 0.9896
OE, *n* (%)	6.67 (0.56, 79.29) 0.1332	9.99 (0.74, 134.46) 0.0827	6.10 (0.38, 98.88) 0.2035
UE, *n* (%)	2.86 (0.67, 12.20) 0.1562	2.51 (0.58, 10.92) 0.2191	1.51 (0.31, 7.32) 0.6104
Onset to admission time, min	1.00 (1.00, 1.00) 0.4765	1.00 (1.00, 1.00) 0.5112	1.00 (1.00, 1.00) 0.4636
rLMC	0.94 (0.86, 1.03) 0.1659	0.96 (0.87, 1.06) 0.4097	1.05 (0.94, 1.18) 0.3931
Laboratory parameters			
NATG	257.59 (1.38, 48103.06) 0.0375*	388.09 (1.92, 78608.77) 0.0278*	1534.87 (5.02, 469638.44) 0.0120*
NLR	1.04 (0.98, 1.10) 0.2453	1.05 (0.98, 1.12) 0.1532	1.01 (0.94, 1.10) 0.7211
SII	1.00 (1.00, 1.00) 0.6638	1.00 (1.00, 1.00) 0.5925	1.00 (1.00, 1.00) 0.6716
PLR	1.00 (0.99, 1.00) 0.6717	1.00 (0.99, 1.00) 0.6159	1.00 (0.99, 1.00) 0.1847
LMR	0.96 (0.81, 1.14) 0.6526	0.95 (0.78, 1.15) 0.6080	0.98 (0.85, 1.14) 0.8398
NAR	19.39 (0.47, 797.17) 0.1179	19.16 (0.36, 1030.43) 0.1465	15.96 (0.21, 1216.70) 0.2104
PNI	1.02 (0.94, 1.10) 0.6327	1.03 (0.95, 1.11) 0.5084	1.00 (0.93, 1.09) 0.9144
dNLR	0.99 (0.89, 1.09) 0.7756	0.97 (0.81, 1.16) 0.7100	0.95 (0.77, 1.17) 0.6118
ALBI	0.80 (0.32, 1.97) 0.6287	0.74 (0.30, 1.82) 0.5116	0.83 (0.34, 2.04) 0.6893
PALBI	1.60 (0.27, 9.31) 0.6021	1.49 (0.24, 9.25) 0.6699	1.21 (0.36, 4.07) 0.7531
LDL‐C/lymphocyte	0.96 (0.69, 1.33) 0.8104	0.96 (0.68, 1.34) 0.8028	0.95 (0.67, 1.34) 0.7578
Platelet, ×109/L	1.00 (0.99, 1.00) 0.3894	1.00 (0.99, 1.00) 0.2947	0.99 (0.99, 1.00) 0.2017
Hb, g/L	1.00 (0.97, 1.02) 0.7278	1.01 (0.98, 1.03) 0.6501	1.01 (0.98, 1.04) 0.4981
INR	1.13 (0.10, 13.04) 0.9227	1.18 (0.09, 15.58) 0.9000	0.15 (0.00, 7.28) 0.3348
DD, mg/L	1.09 (1.00, 1.20) 0.0561	1.11 (1.01, 1.22) 0.0345*	1.12 (1.01, 1.25) 0.0249*
TC, mmol/L	1.00 (1.00, 1.01) 0.1235	1.00 (1.00, 1.01) 0.1208	1.01 (1.00, 1.01) 0.0280*
TG, mmol/L	1.03 (0.97, 1.10) 0.3195	1.04 (0.97, 1.11) 0.2657	1.05 (0.97, 1.13) 0.2346
LDL, mmol/L	0.65 (0.39, 1.07) 0.0877	0.63 (0.38, 1.04) 0.0723	0.79 (0.46, 1.38) 0.4111
HDL, mmol/L	1.63 (0.83, 3.20) 0.1556	1.56 (0.76, 3.21) 0.2286	1.70 (0.71, 4.07) 0.2307
Serum glucose, mmol/L	1.00 (0.97, 1.02) 0.8181	1.00 (0.96, 1.03) 0.7954	1.00 (0.95, 1.05) 0.8481
Albumin, g/L	1.02 (0.95, 1.10) 0.5913	1.03 (0.95, 1.11) 0.4657	1.01 (0.93, 1.09) 0.8202
Uric acid, μmol/L	1.00 (0.99, 1.00) 0.4163	1.00 (1.00, 1.00) 0.6521	1.00 (0.99, 1.00) 0.5312
Creatinine, μmol/L	1.00 (0.98, 1.01) 0.5653	1.00 (0.98, 1.01) 0.8770	0.99 (0.97, 1.02) 0.6655
Treatment after admission			
Antiplatelet	0.49 (0.18, 1.35) 0.1673	0.48 (0.17, 1.35) 0.1640	0.89 (0.22, 3.55) 0.8665
Anticoagulant	0.77 (0.22, 2.75) 0.6890	0.71 (0.19, 2.61) 0.6008	0.50 (0.11, 2.32) 0.3765
Statins	0.27 (0.09, 0.77) 0.0148*	0.27 (0.09, 0.79) 0.0165*	0.53 (0.16, 1.75) 0.2973
Antihypertensives	2.81 (1.13, 7.01) 0.0264*	2.52 (0.99, 6.40) 0.0523*	2.67 (0.93, 7.69) 0.0682

Abbreviations: AIS = Acute ischemic stroke; ALBI = albumin–bilirubin; BMI=body mass index; CE = cardioembolic; CI=confidence interval; DBP=diastolic blood pressure; DD=D‐dimer; dNLR = derived neutrophil‐to‐lymphocyte ratio; HDL = high‐density lipoprotein; II = systemic immune‐inflammation index; INR = international normalized ratio; LAO = Large vessels atherosclerosis; LDL = low‐density lipoprotein; LMR = lymphocyte‐to‐monocyte ratio; NAR = neutrophil‐to‐albumin ratio; NATG = NAR–triglyceride ratio; NIHSS=National Institute of Health Stroke Scale; NLR = neutrophil‐to‐lymphocyte ratio; SOE = other etiology; OR = odds ratio; PALBI = platelet–albumin–bilirubin; PLR = platelet–lymphocyte ratio; PNI = prognostic nutritional index; rLMC = regional leptomeningeal score; SAO=small‐artery occlusion; SBP=systolic blood pressure; TC = total cholesterol; TG = triglyceride; TOAST = Trial of ORG 10172 in Acute Stroke Treatment; UE = undetermined etiology.

^a^ Model 1 adjusted for age and gender.

^b^ Model 2 adjusted for Model 1 adding to baseline NIHSS, TOAST, anticoagulant history, statins after admission, and antihypertension after admission.

* *p* value less than 0.05.

Subsequently, we conducted Youden index and AUC‐ROC to determine the optimal cut‐off threshold for those continuous independent risk factors to distinguish between sHT and non‐sHT (Figure 2C). The best cut‐off values for these risk factors were 0.059 for the NATG, 13 for the baseline NIHSS, 3.6 for TC, and 0.86 for DD, respectively (Figure 2D).

### 3.4 Development and Validation of the Nomogram

**FIGURE 3:**
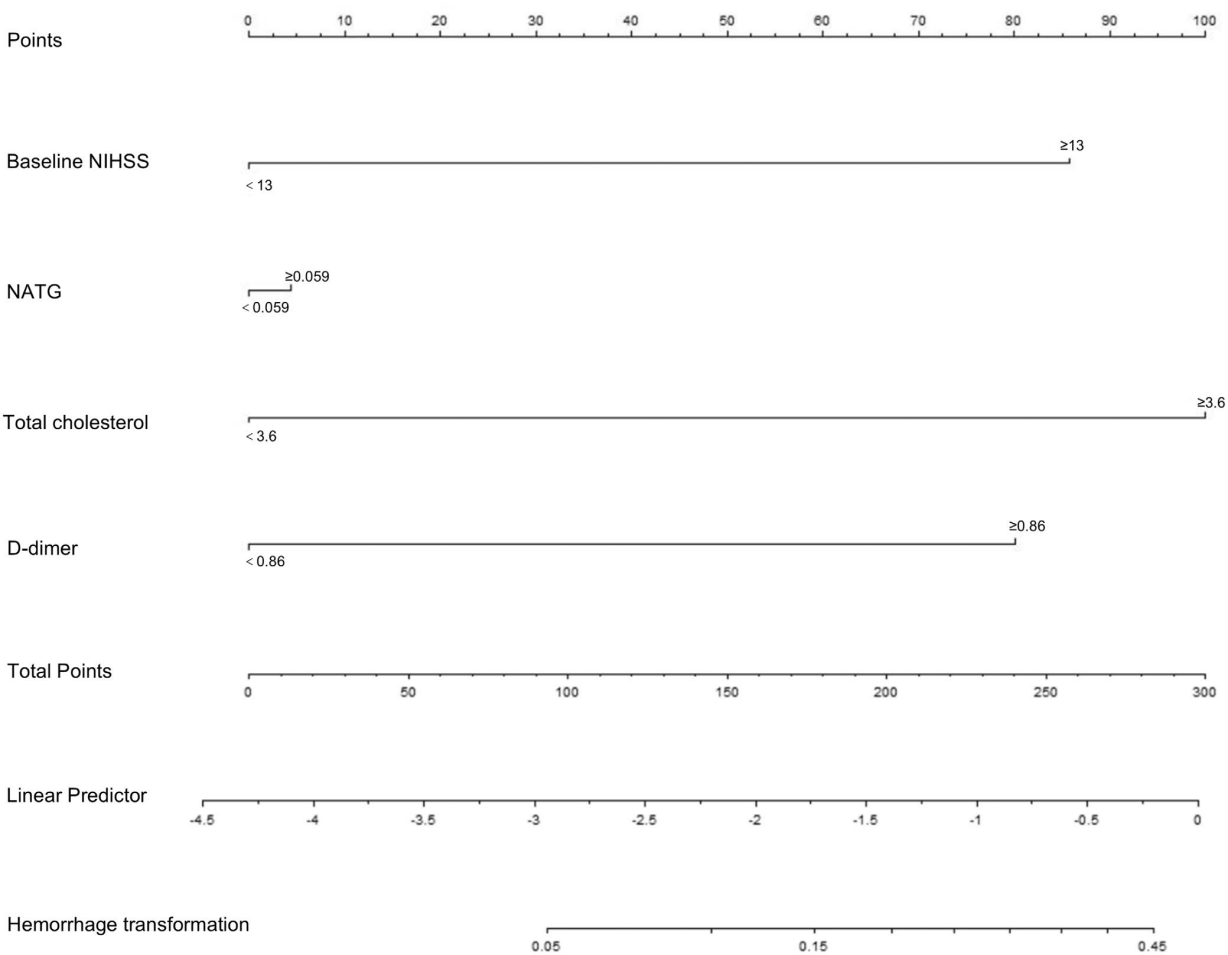
The novel indicator integrating NATG and other clinical features was developed into nomogram. NATG = NAR–triglyceride ratio; NIHSS = National Institute of Health Stroke Scale; TOAST = Trial of ORG 10172 in Acute Stroke Treatment.

The training cohort (228 patients) was included in the study for establishing the nomogram. Variables with significant statistical differences in the multivariable logistic regression, including baseline NIHSS ≥ 13, novel index ≥ 0.059, TC ≥ 3.6, and DD ≥ 0.86, were selected for developing the nomogram. As shown in Figure 3, the total score was calculated by adding the corresponding score of each risk factor to estimate the probability of sHT.

**FIGURE 4:**
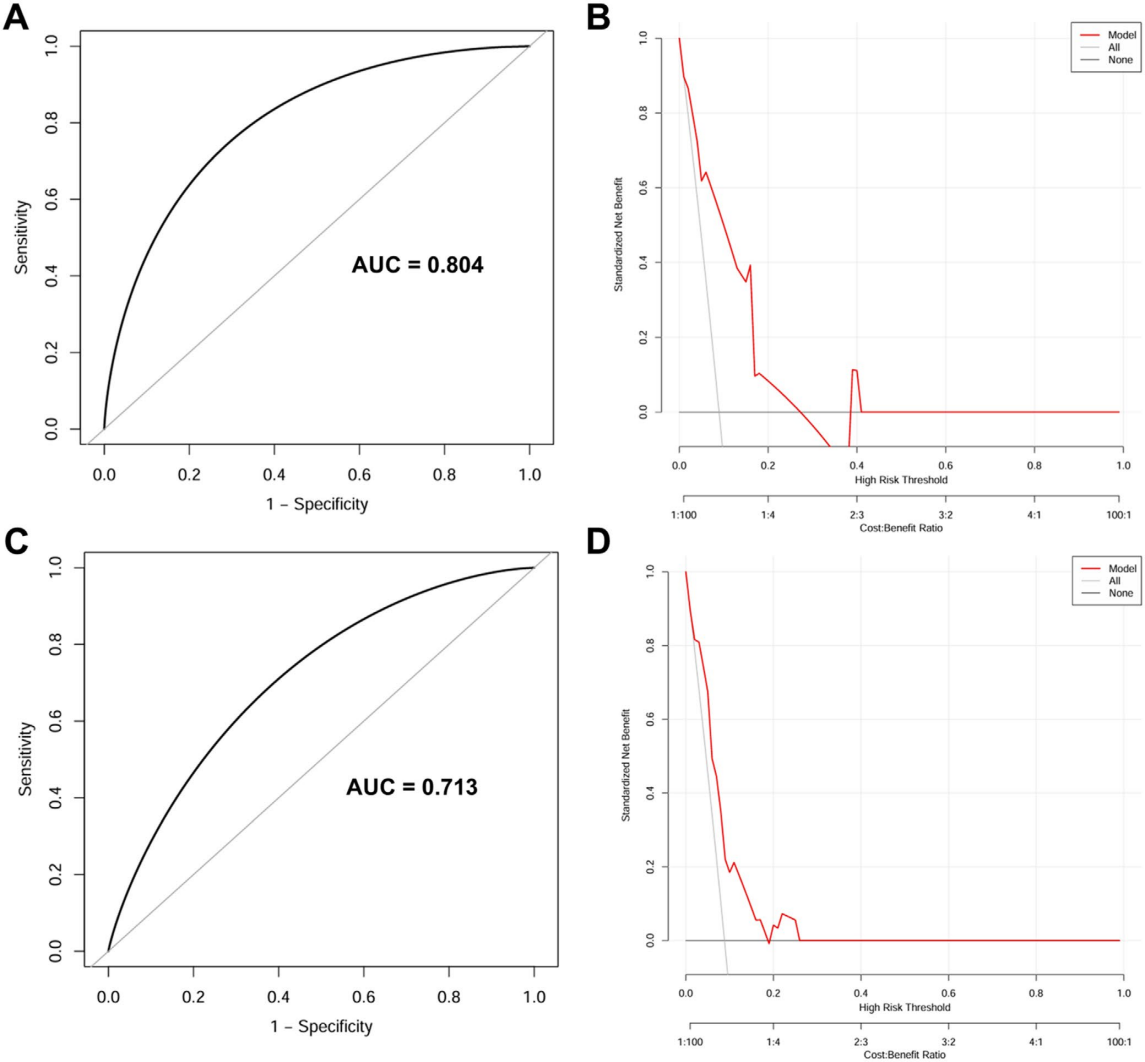
The performance of the nomogram model. (A, B) The training cohort. (C, D) The validation cohort. (A, C) Calibration curves suggest that our nomogram performed well in both derivations. (B, D) The DCA curves show that our nomogram had good practicability in both internal and external validation. AUC = area under the ROC curve; DCA = decision curve analysis; ROC = Receiver operating characteristic.

The calibration plots using 500 bootstrapping resamples displayed a good consistency of the model in both the internal (0.804, 95% CI: 0.643–0.918) and external validation (0.713, 95% CI: 0.499–0.868), as shown in Figure 4A,C. Moreover, DCA was performed to estimate the clinical usefulness, with the net benefit rate plotted on the *y*‐axis and the high‐risk threshold on the *x*‐axis, where the high‐risk threshold was set at 0.1. As shown in Figure 4B,D, the DCA demonstrated good practicality in both internal and external validation.

## 4 Discussion

HT is a severe complication of AIS with a significant impact on patients' prognosis. Accumulating evidence suggests that inflammation plays a crucial role in the pathophysiology of AIS and thrombosis. Furthermore, inflammation and immune system activation are likely key contributors to blood–brain barrier (BBB) disruption in the pathophysiology of HT [5]. sHT can be challenging in early identification and detection owing to its often asymptomatic or without warning signs [26]. Several inflammatory biomarkers derived from CBC and blood biochemistry have been reported that are related to occurrence of HT and may have predictive or prognostic value for HT. Although the role of these inflammatory biomarkers in predicting HT risk following IVT or EVT in AIS patients has been extensively investigated, there is still a lack of understanding of their effects on sHT, given that predictors of sHT differ from those in HT following IVT [14, 26, 27, 28]. Moreover, inflammatory biomarkers could be promising as significant predictors of sHT, potentially aiding in clinical decision‐making and monitoring prognosis during the acute stage [26]. Therefore, this study aimed to determine the independent predictor within inflammatory biomarkers and to develop a novel inflammatory index. Furthermore, we establish a nomogram model based on NATG and other significant risk factors for convenient usefulness in clinical practice.

In the present study, sHT was observed in 31 of 325 patients (9.5%) over a 3‐month period after AIS onset, which was similar to previous studies (9.6%–12.3%) [26, 29]. The novel index combined with NAR and TG was positively associated with the risk of sHT in AIS patients and the significance relationship was further supported in the validation cohort. Importantly, the predictive power of NATG for sHT was superior to that of the other inflammatory biomarkers. As for NAR, our previous study found that NAR levels have stated a negative relationship of 3‐month functional outcomes in AIS patients. Moreover, a prior study found that NAR could be recognized as an independent risk factor for aneurysmal subarachnoid hemorrhage [30]. The NAR, defined as the neutrophil counts divided by the serum albumin, is the result of increasing neutrophil and/or decreasing serum albumin level [31]. Specifically, elevated neutrophil counts represented an inflammatory activity that could injure vascular and cause tissue ischemia [32]. It is noted that increasing neutrophil also has a profound impact on plaque development and instability which may motivate the platelet aggregation [33]. Regarding TG, several previous studies demonstrated that there was a correction between serum TG level and cerebral hemorrhage through undermining cerebral vessel completeness [34, 35]. Moreover, we also evaluated the interaction between NAR and TG and found no significant effect. Based on above, it is reasonable that integrating NAR and TG into a novel indicator (NATG) has demonstrated potential importance for predicting the occurrence of sHT with AIS in our nomogram. Further confirmations in different populations and larger sample sizes should be conducted in future.

Additionally, DD has been identified as predictor of sHT in our nomogram model. Both are coagulation and fibrinolysis parameters. Obviously, the coagulative function plays pivotal role in hemorrhagic diseases, including HT in AIS [36]. DD is a product of fibrin degradation by plasmin, and the elevating DD level indicates a hypercoagulable status [37, 38]. In accordance with previous studies, we found that elevating DD was associated with a higher sHT risk [39, 40]. Specifically, DD level greater than 0.86 mg/L was identified as the optimal cut‐off value for predicting sHT risk in this study. In brief, DD is an independent indicator of the sHT occurrence.

Consistent with previous studies [9, 10, 14], we found that the baseline NIHSS was a significant predictor of sHT in AIS patients. Notably, the NIHSS is the most critical predictor in our nomogram model. It is known that the higher the NIHSS, the more severe the degree of neurological deficiency and the greater the susceptibility to HT in AIS patients [41]. Although poor collateral circulation has been reported as a predictor of HT in previous studies [13, 41, 42], there was no statistically significant association between collateral status (rLMC) and the occurrence of sHT in our study. The possible reasons may be due to most previous studies focusing on the risk of HT in AIS patients undergoing recanalization treatments [41, 42], whereas our study aimed to identify the risk factors of sHT in AIS patients without thrombolysis and thrombectomy treatments. Another reason could be the limited sample size of our study. Furthermore, we would consider more effective imaging markers of collateral status to predict the risk of sHT in AIS patients, and larger‐scale investigations may be warranted to clarify these results and confirm their potential clinical value.

The main strength of this study is the identification of NATG—a novel indicator derived from inflammatory biomarkers and serum lipids, and the construction of a nomogram model for clinical application. Moreover, our nomogram demonstrated significant predictive capability, achieving an AUC of 0.804. However, some limitations of our study should be noted. Firstly, given our study is a single‐center, retrospective observational design with a relatively small sample size, it inevitably contains biases, including selection bias. Secondly, according to ECASS criteria, HT could be classified into HI or PH subtypes. However, we did not perform statistical analysis on each HT subgroup, considering the limited sample size and need for sufficient statistic power. Thirdly, we collected blood samples within the first 24 h after admission, which limits the dynamic observation of changes in inflammatory indices. Future studies should involve larger cohorts, prospective data collection, and multiple centers to address these limitations.

In conclusion, this study found a novel combined indicator, NATG, that was significantly positively related to sHT occurrence in AIS patients. A nomogram model based on NATG and other important risk factors was constructed for clinical use, which is beneficial for accurately identifying individuals at high risk of sHT and enabling timely prevention and therapeutic.

## Author Contributions

J.B., J.F., and L.H. researched the literature and conceived the study. J.B., M.M., and K.W. were involved in protocol development, gaining ethical approval, and patient recruitment. J.B., M.M., J.W., M.Z., J.G., and N.C. conducted the data analysis and interpretation. J.B. and M.M. wrote the first draft of the manuscript. All authors reviewed and edited the manuscript and approved the final version of the manuscript.

## Ethics Statement

The Ethical Review Committee of the West China Hospital of Sichuan University approved this research (No. 2020(069)).

## Consent

All authors agreed to submit the paper.

## Conflicts of Interest

The authors declare no conflicts of interest.

## Supporting information


Table S1.

## Data Availability

The datasets used and/or analyzed during the current study are available from the corresponding author upon reasonable request.
