# Supplementary material for: Integrating Neutrophil‐To‐Albumin Ratio and Triglycerides: A Novel Indicator for Predicting Spontaneous Hemorrhagic Transformation in Acute Ischemic Stroke Patients
Source: CNS Neurosci Ther. 2024 Dec 17;30(12):e70133. doi: 10.1111/cns.70133 (PMC11652394; doi:10.1111/cns.70133)
Supplement: Supplementary file 1 — Table S1. [file CNS-30-e70133-s001.docx]

|  |  | **NAR**^†^ | | | **P for interaction** |
| --- | --- | --- | --- | --- | --- |
|  |  | **low**(~-0.092) | **middle**(0.093-0.180) | **high**(0.181-~) |  |
| **TG**^†^ | |  |  |  |  |
| Low (~-0.92) | | - | 2.52 (0.46, 13.83) 0.2872 | 1.11 (0.14, 8.64) 0.9240 | 0.5328 |
| middle(0.93-1.80) | | 0.00 (0.00, Inf) 0.9905 | 1.05 (0.13, 8.18) 0.9629 | 0.25 (0.00, 5365.30) 0.7843 |  |
| high(1.81-~) | | 0.91 (0.12, 7.07) 0.9306 | 1.83 (0.30, 11.02) 0.5114 | 0.87 (0.07, 10.69) 0.9167 |  |

**Table S1.** Interaction analysis between NAR and TG in sHT among AIS patients.

^†^: Results are presented as OR (95% CI), with corresponding p-values

NAR=neutrophil-to-albumin ratio; TG=triglyceride; AIS=acute ischemic stroke; OR=Odds Ratio; CI=Confidence Interval; sHT=spontaneous hemorrhagic transformation.
